# Enzymatic Reaction Network‐Driven Polymerization‐Induced Transient Coacervation

**DOI:** 10.1002/anie.202421620

**Published:** 2024-12-23

**Authors:** Surbhi Sharma, Andrea Belluati, Mohit Kumar, Shikha Dhiman

**Affiliations:** ^1^ Department of Chemistry Johannes Gutenberg University Mainz Duesbergweg 10–14 Mainz 55122 Germany; ^2^ Department of Chemistry and Centre for Synthetic Biology Technical University of Darmstadt Peter-Grünberg-Straße 4 Darmstadt 64287 Germany

**Keywords:** BioATRP, Liquid-liquid phase separation, Enzymatic Reaction Network, Coacervates, ATP

## Abstract

A living cell has a highly complex microenvironment whereas numerous enzyme‐driven processes are active at once. These procedures are incredibly accurate and efficient, although comparable control has not yet been established in vitro. Here, we design an enzymatic reaction network (ERN) that combines antagonistic and orthogonal enzymatic networks to produce adjustable dynamics of ATP‐fueled transient coacervation. Using horseradish peroxidase (HRP)‐mediated Biocatalytic Atom Transfer Radical Polymerization (BioATRP), we synthesized poly(dimethylaminoethyl methacrylate), which subsequently formed coacervates with ATP. We rationally explored enzymatic control over coacervation and dissolution, using orthogonal and antagonistic enzyme pairs *viz*., alkaline phosphatase, Creatine phosphokinase, hexokinase, esterase, and urease. ATP‐fuelled coacervates also demonstrate the enzymatic catalysis to prove its potential to be exploited as a cellular microreactor. Additionally, we developed ERN‐polymerization‐induced transient coacervation (ERN‐PIC), with complete control over the system, polymerization, coacervation, and dissolution. Notably, the coacervation process itself determines functional properties, as seen in selective cargo uptake. The strategy offers cutting‐edge biomimetic applications, and insights into cellular compartmentalization by bridging the gap between synthetic and biological systems. The development of temporally programmed coacervation is promising for the spatial arrangement of multienzyme cascades, and offers novel ideas on the architecture of artificial cells.

## Graphical abstract

An enzymatic reaction network (ERN) for polymerization‐induced transient coacervation (ERN‐PIC), with complete control over the system: polymerization, coacervation, dissolution and selective cargo uptake.

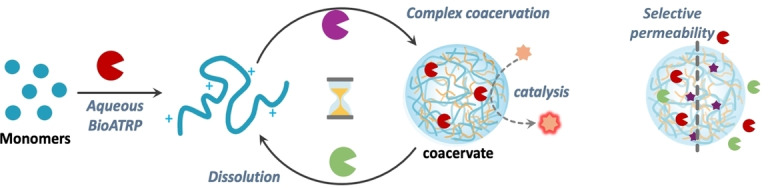

## Introduction

Compartmentalization is a hallmark of biological systems, essential for controlling a plethora of processes within cells.[^1^, ^2^] Generally, compartmentalization is associated with cellular structures, delineated by lipid membranes, such as the nucleus, mitochondria, and Golgi body.[^3^, ^4^] However, an emerging interest lies in membraneless organelles (MLOs) formed through complex coacervation—a process where dynamic, liquid‐like structures arise from interactions among charged polyelectrolytes, such as proteins and nucleic acids.[^5^, ^6^, ^7^] These MLOs continuously undergo internal rearrangement and exchange of components with their environment. This dynamic behavior enables them to form and dissolve in response to biochemical signals.

Efforts to replicate life‐like behaviors in synthetic systems are actively progressing.[^2^, ^4^, ^8^, ^9^] Studies have explored enzymatic control for reversible coacervation‐dissolution process;[^10^, ^11^, ^12^, ^13^] for instance, pyruvate kinase, and hexokinase have been used to regulate adenosine tri‐ & di‐phosphate (ATP and ADP) levels, thereby controlling peptide‐based coacervate formation and dissolution by Spruijt's, and Keating's group.[^14^, ^15^, ^16^] Dekker, and co‐workers utilized a kinase/phosphatase enzyme pair to modulate the phosphorylation states of peptides, leading to reversible RNA‐peptide coacervation.^[17]^ Additionally, Huck, and co‐workers used oscillating trypsin concentrations to manage the assembly and disassembly of polyglutamic acid and lysine‐serine coacervates.^[18]^ Martin, and co‐workers, and Bishop, Obermeyer and co‐workers independently designed enzyme/polyelectrolyte coacervates where the enzyme was a participant in coacervation as well as responsible for its dissolution.[^19^, ^20^] Dogterom and Reese revealed that RNA/spermine‐based coacervates display transient nonspherical shapes during enzymatic reactions.^[21]^ Walther and co‐workers developed programmable ATP‐Fueled DNA Coacervates using DNA‐based enzymatic reaction networks (ERN).[^22^, ^23^] Maiti and co‐workers used ERN to temporally control the conformational disposition of a protein in a condensate state.^[24]^ Most synthetic coacervates are composed of polymers (e.g., polysaccharides, nucleotides) that interact with a counter polymer or small molecule.[^25^, ^26^, ^27^, ^28^] However, achieving coacervation with small molecules remains challenging[^29^, ^30^, ^31^, ^32^, ^33^, ^34^, ^35^] due to the delicate balance required between intermolecular interactions and entropic penalties. Therefore, alternative strategies are needed for synthesizing coacervates from small molecules in aqueous environments.

**Scheme 1:**
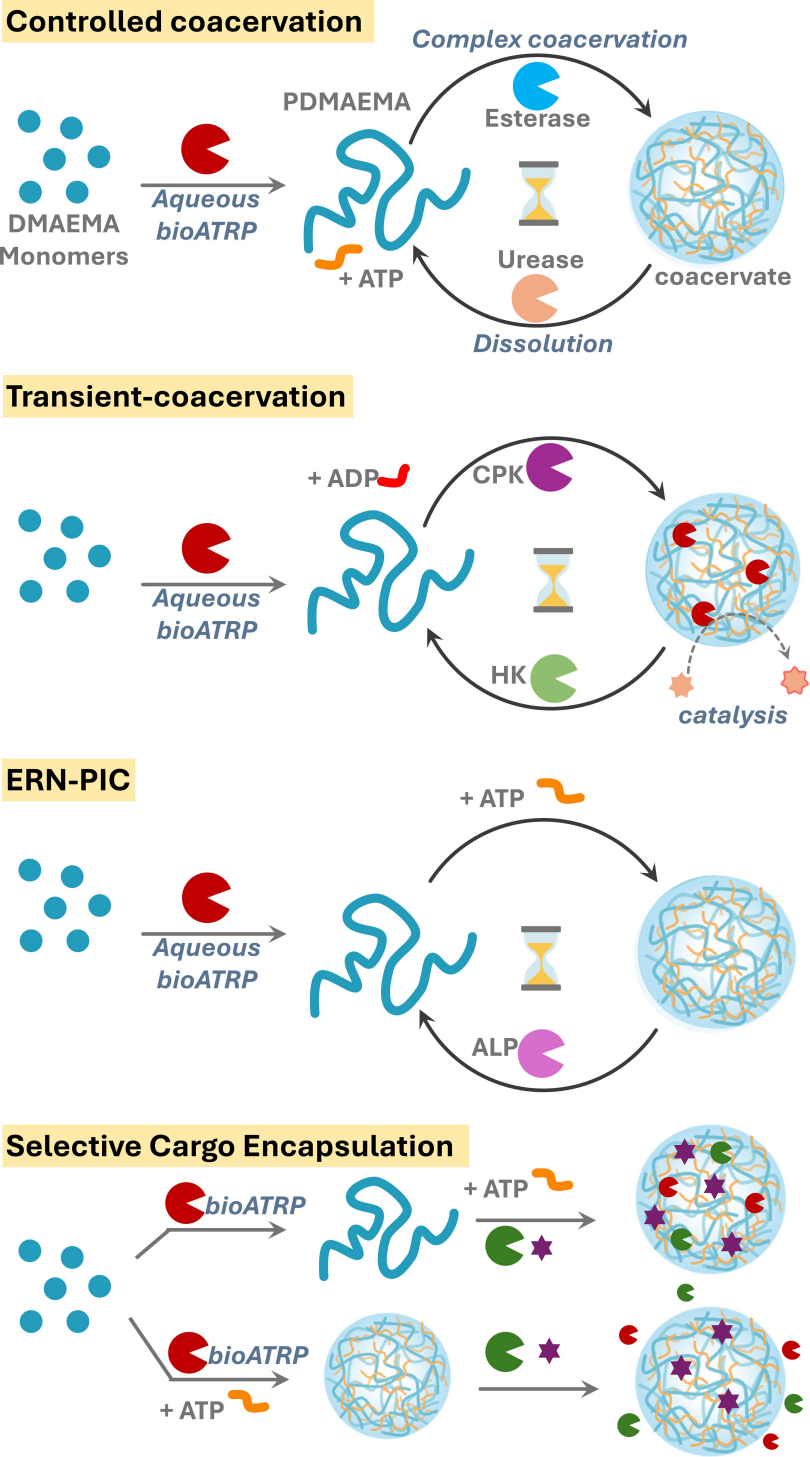
Schematic illustration of enzymatic reaction networks (ERNs) driving in situ polymer synthesis using aqueous BioATRP, time‐controlled complex coacervation with ATP, and dissolution. The coacervates also depict microreactor behavior and selective cargo encapsulation.

One promising approach is Biocatalytic Atom Transfer Radical Polymerization (BioATRP), where metalloenzymes such as horseradish peroxidase, catalase, hemoglobin, and myoglobin (Mb), initiate and control radical polymerizations.[^36^, ^37^, ^38^, ^39^] In this study, we developed an ERN that not only controls the transient coacervation process but also enables the in situ synthesis of coacervate components—the formation of polymers from small molecule precursors (Scheme 1). This approach mirrors the natural synthesis of intrinsically disordered proteins from simple amino acids and the proteins subsequently undergo coacervation. This biomimetic strategy, which enables polymer synthesis directly from small molecules without the need for complex organic synthesis or purification steps, offers a simplified pathway to coacervate formation. Using BioATRP, we synthesized length, and dispersity‐controlled polymers of 2‐(Dimethylamino)ethyl methacrylate (DMAEMA). This polymer PDMAEMA undergoes polymerization‐induced coacervation at pH below its pKa, in the presence of a counter small molecule ATP. The dependence of coacervation on environmental pH, and counter ion ATP are further exploited in different ERNs to temporally program coacervation using pH and phosphorylation. Furthermore, the coacervates demonstrate their selective and contrasting encapsulation capability for various enzymes as well as small molecules based on in situ and ex situ preparation methods, which provided us with opportunity to develop these coacervates as microreactors (Scheme 1).

## Results and Discussion

### Design Strategy

**Figure 1:**
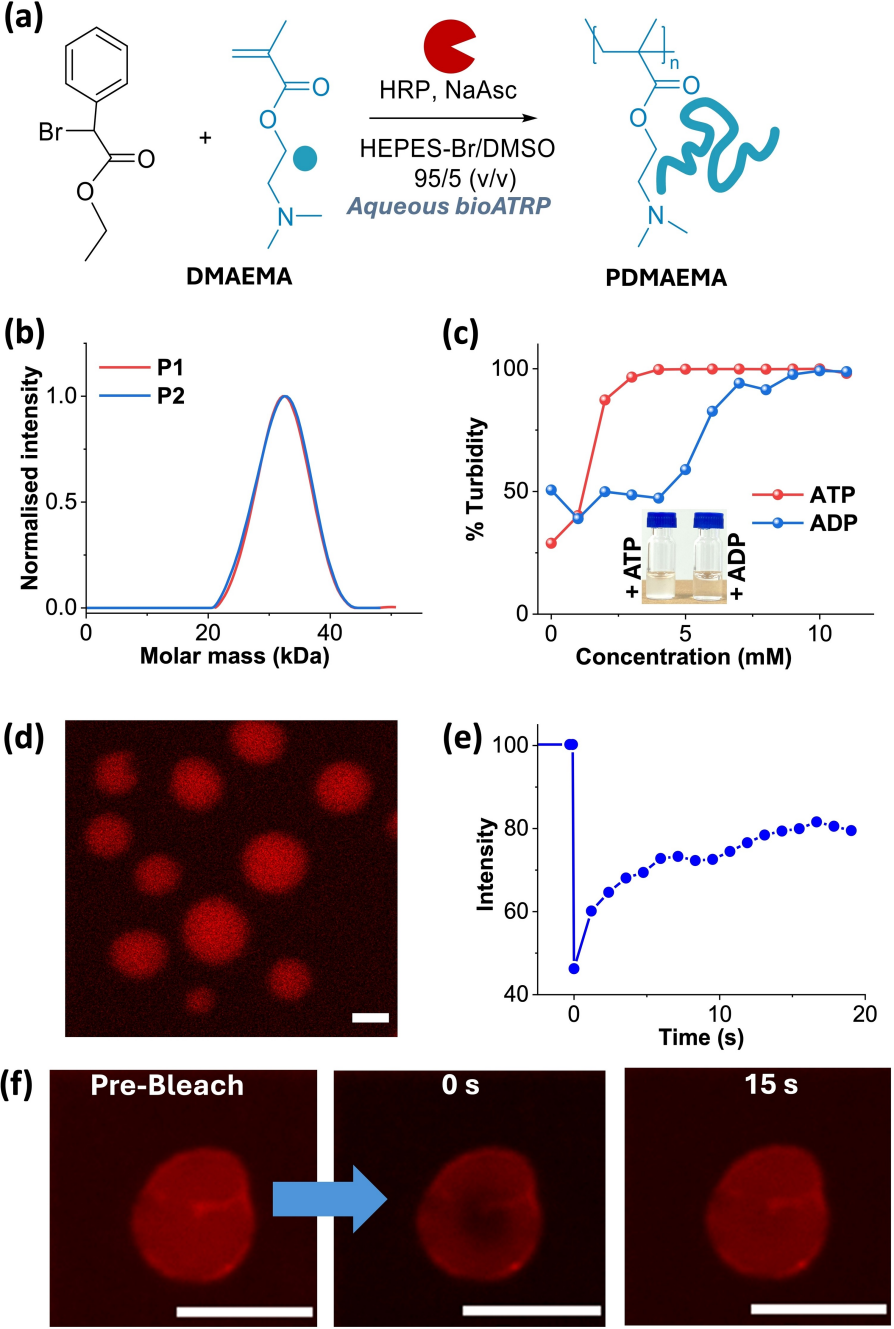
(a) Scheme for PDMAEMA synthesis through BioATRP. (b) Molar mass distribution of polymers P1 and P2 synthesized in two different batches. (c) Turbidity measurements for titration of PDMAEMA with ATP and ADP. (d) CLSM images of complex coacervates of PDMAEMA with ATP, [Resorufin]=0.5 mol %, scale bar=2 μm. (e) FRAP kinetics. (f) Corresponding CLSM images obtained during FRAP of PDMAEMA with 5 mM ATP coacervate, [Nile red]=0.5 mol %, scale bar=5 μm. [PDMAEMA]=0.5 mM, 50 mM HEPES, pH 7.3

We first synthesized polymer of 2‐(dimethylamino) ethyl methacrylate (PDMAEMA) using the BioATRP approach with metalloenzyme Horseradish Peroxidase (HRP) to control the polymerization (Figure 1a). Polymers of 20–40 kDa, with a degree of polymerization of ~200 and low dispersity ~1.1 is yielded (P1=batch 1, P2=batch 2) (Figure 1b, Table S1). Despite moderate monomer conversion (57–61 %, Figure S1), unreacted monomers, do not affect subsequent studies due to their incapability to coacervate. PDMAEMA was used without further purification.

### Turbidity‐Based Titrations of Polymer with ATP and ADP

The pKa of the amine group in PDMAEMA was determined by acid‐base titration to be 7.4 (Figure S2).^[40]^ To assess the phase‐separation behavior of PDMAEMA in aqueous conditions, we adjusted the pH of the PDMAEMA solution to 7.3 using 50 mM HEPES buffer. This pH near physiological levels was chosen to optimize enzyme activity and to improve the biological relevance of these coacervates for potential applications. At this pH, PDMAEMA remains partially protonated, however, its multivalent character provides sufficient cationic charge to interact effectively with negatively charged multivalent guest molecules. To investigate the coacervation potency of the resultant cationic polymer, we selected adenosine triphosphate (ATP) as a guest for complex coacervation with PDMAEMA because of its multivalent nature,^[41]^ where above pH 6.5 it bears a −4 charge, and below this pH, it bears a −3 charge.^[42]^ Moreover, due to its biorelevant feature, it can be synthesized or consumed by different enzymes,[^43^, ^44^] which can provide us additional control over the coacervation process consumed by different enzymes,[^43^, ^44^] which can provide us additional control over the coacervation process. Generally, enzymatic reactions of ATP are its conversion to or synthesis from adenosine diphosphate (ADP) through phosphorylation‐dephosphorylation reactions.[^14^, ^45^]

Since ADP is also a multivalent guest and perhaps can have the ability to undergo complex coacervation with PDMAEMA. Hence, it was necessary to compare the coacervation of PDMAEMA with both ATP and ADP. For this, we conducted turbidity‐based titrations by incrementally adding ATP and ADP from (0–10 mM) to 0.5 mM PDMAEMA solution (Figure 1c). The changes were monitored by measuring absorbance at 600 nm, where neither the component of the solution nor the polymer absorbs, which is converted into % Turbidity. The onset of turbidity, and saturation occurred at lower concentrations for ATP compared to ADP, due to its higher charge density. To investigate the origin of turbidity, we used confocal laser scanning microscopy (CLSM) to visualize the structures of PDMAEMA alone, as well as in the presence of 5 mM ATP, and 5 mM ADP separately (Figure 1d, S3). The samples were visualized in solution state by conventional dye encapsulation method using an external fluorescent probe (0.5 mol % of Nile Red or Resorufin). The encapsulation capability of PDMAEMA‐ATP is confirmed by using two different dyes, and corresponding CLSM images (Figure 1d,f). We observed small irregular aggregates of polymer alone and with ADP. In contrast, spherical structures formed in the presence of ATP exhibited rapid fluorescence recovery after photobleaching (FRAP) (Figure 1e,f, and S4). This confirms the formation of complex coacervates. Therefore, for further studies, we proceeded with 5 mM guest concentration, where only ATP forms coacervates. The reversibility, and responsiveness of this coacervation were confirmed by changing the pH of the solution (Figure S5).

### pH‐Responsive Coacervation‐Dissolution with Two Enzymes

**Figure 2:**
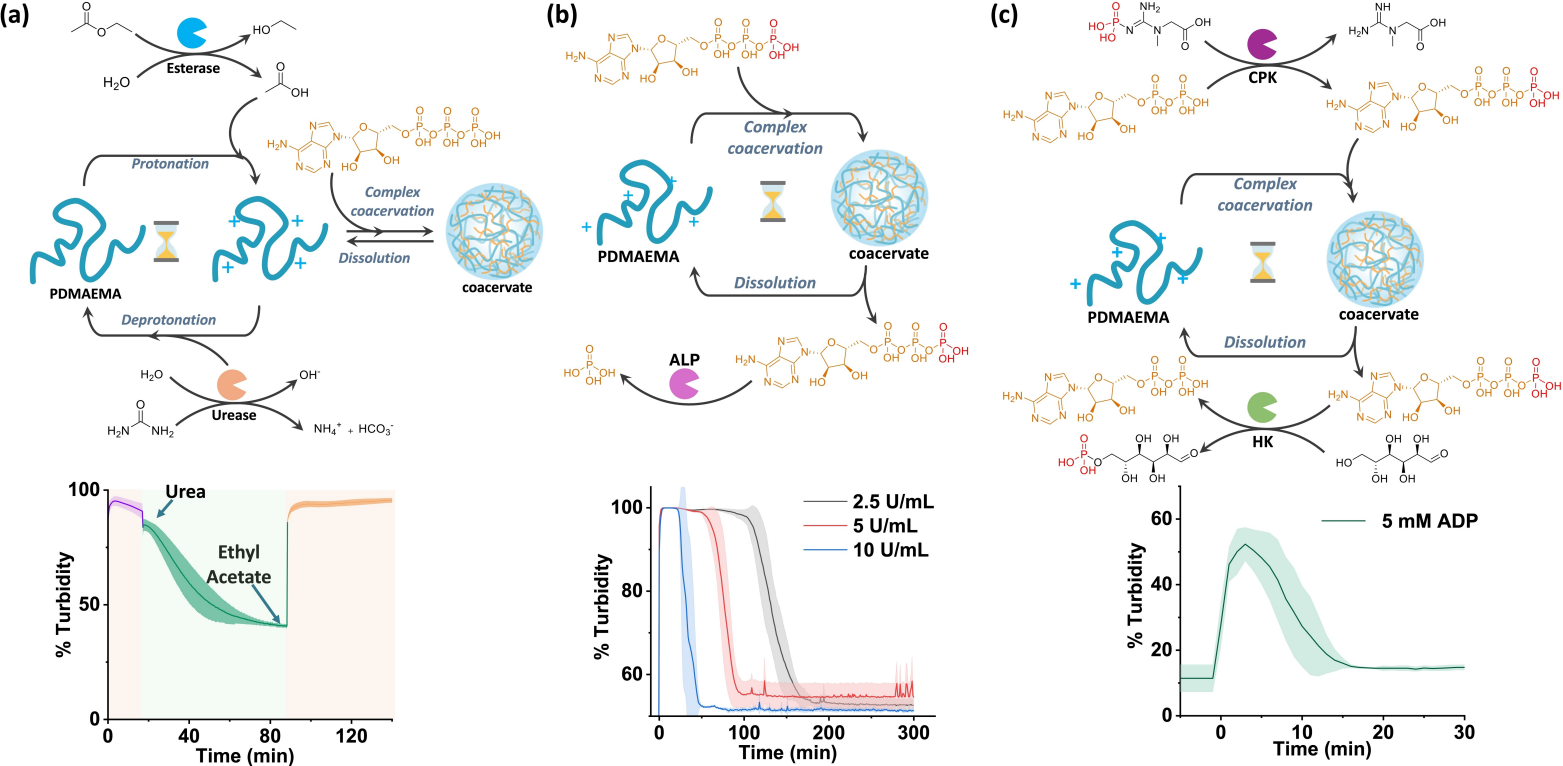
(a) Enzymatically mediated stimuli responsive coacervation. Kinetics of the pH‐modifying enzymes. Arrows indicate the addition of substrates. [PDMAEMA]=1.25 mM, [ATP]=8 mM, Esterase=200 U/mL, Urease=285 U/mL, [ethyl acetate]=240 mM, [urea]=24 mM, and 4.5 pH, 10 mM acetate buffer. (b) ATP‐driven ALP‐mediated transient coacervation of PDMAEMA. Time dependent % turbidity changes with varied units of ALP. [PDMAEMA]=0.5 mM, [ATP]=5 mM, 50 mM HEPES, pH 7.3. (c) ERN‐mediated ATP‐driven transient coacervation of PDMAEMA (HK, and CPK). Time‐dependent % Turbidity changes. [PDMAEMA]=0.5 mM, [ATP]=[ADP]=5 mM and 4 mM, CPK=20 U/mL, HK=10 U/mL, [PCr]=7.5×10^−3^ M, [Glucose]=0.1 M, 50 mM HEPES and pH 7.3. N=3,±standard deviation.

After confirming the reversible formation of complex coacervates, we establish a reversible coacervation‐dissolution process using two antagonistic enzymes: esterase and urease (Figure 2a). Urease catalyzes urea hydrolysis to produce ammonia, raising the pH by consuming protons, while esterase converts ethyl acetate into ethanoic acid, and ethanol, lowering the pH by consuming hydroxide ions. Initially in the acetate buffer at pH 4, protonated polymer complex with ATP to form coacervates, observed as high turbidity. Addition of urea generates in situ ammonia, increasing pH and causing polymer deprotonation, leading to coacervate dissolution. Conversely, adding esterase and ethyl acetate produces ethanoic acid in situ, lowering pH and thereby reinducing coacervation and turbidity (Figure 2a). Time‐dependent CLSM imaging confirmed coacervate dissolution and reformation driven by urease and esterase activity, respectively (Figure S6). However, this process is most effective during the first cycle, as by‐product accumulation and substrate degradation inhibit enzyme activity. A similar pH‐reversible coacervation is observed with urease and glucose oxidase coupling (Figure S7).

### Transient Coacervation with Single Enzyme

After confirming the formation of reversible complex coacervates, we next aim to establish a transient coacervation using ERN. To investigate the transient enzymatic switching of ATP‐fueled coacervates, initially only Alkaline Phosphatase (ALP) was used. ALP catalyses the hydrolysis of phosphate groups from ATP (Figure 2b). To achieve transient coacervation, the rate of coacervation must exceed its rate of dissolution. We adjusted the concentrations of enzymes, and ALP concentrations, introducing 2.5, 5, and 10 U/mL ALP into a 50 mM HEPES pH 7.3 solution with 0.5 mM PDMAEMA. Without ATP, no change in the absorbance or turbidity occurred, indicating no coacervation. Adding 5 mM ATP initiated coacervation, resulting in turbidity increase (Figure 2b).Over time, we observed coacervate dissolution due to ATP hydrolysis by ALP. Additionally, increasing ALP units, accelerated the onset of dissolution, demonstrating temporally regulated coacervation. Corresponding time‐dependent CLSM images (Figure S8) confirmed a decline in coacervate count due to ATP hydrolysis by ALP.

### Transient Coacervation with ATP‐Based ERN

To enhance control over transient coacervation, we designed ERN with two complementary phosphoryl transferases: Creatinine Phosphokinase (CPK), and Hexokinase (HK), and their substrates Phosphocreatine (PCr) and glucose, which drive ATP formation, and hydrolysis (Figure 2c). HK hydrolyzes ATP to ADP, while CPK regenerates ATP from ADP using PCr. As a result, we obtain temporal control over both coacervation and dissolution. We introduced 4 mM ADP to a solution consisting of PDMAEMA, HK, CPK, and glucose. Initially, neither enzyme was active (Figure S9,10). After addition of PCr, a gradual increase in turbidity is observed, indicating an increasing population of coacervates due to in situ synthesized ATP. The coacervation increased continuously for a few minutes (about 10 minutes, see Figure 2c). Thereafter, decay occurred due to HK‐catalyzed hydrolysis of ATP when the primary fuel PCr was completely consumed to (re)generate ATP.

**Figure 3:**
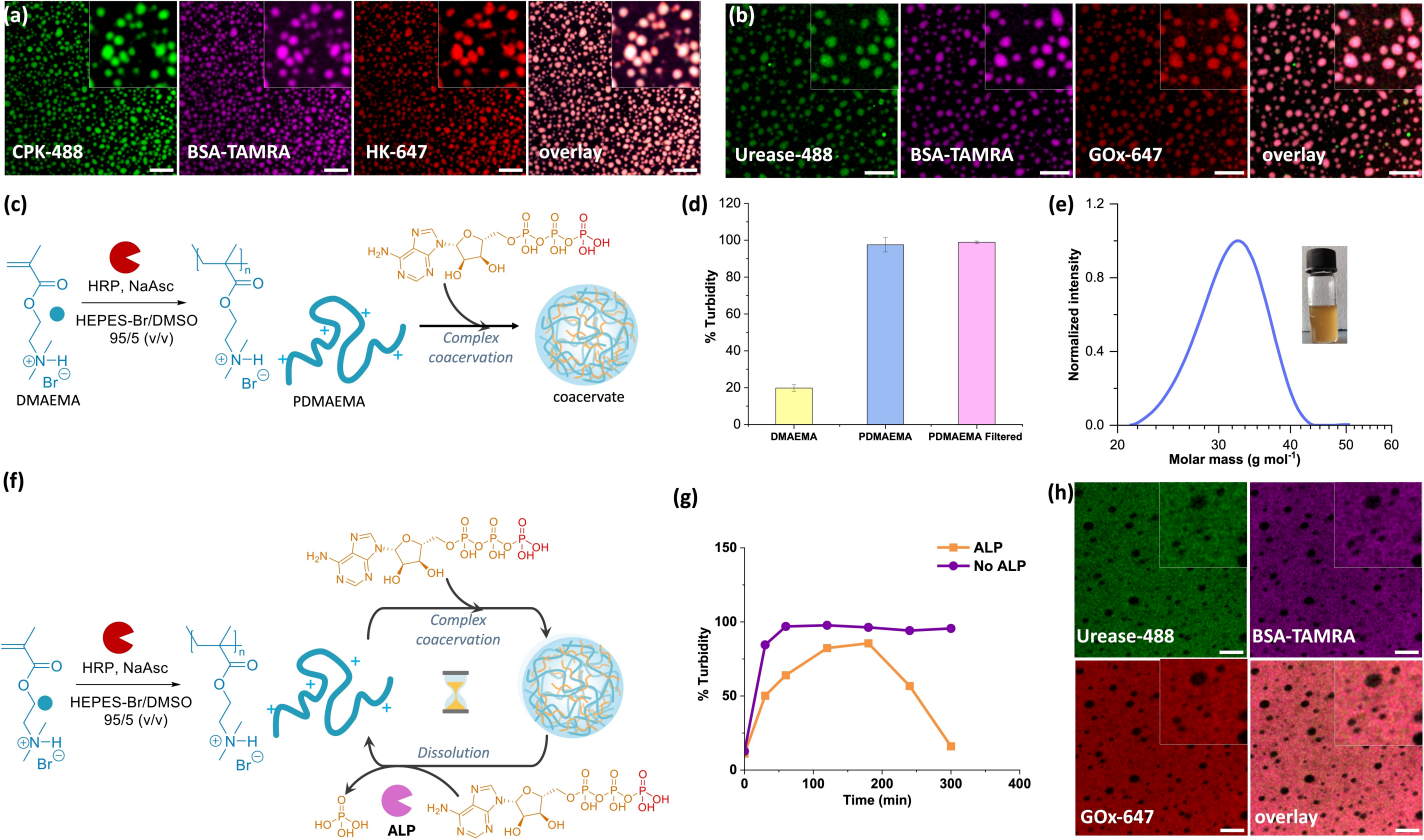
CLSM images of coacervates with segregated protein/enzymes. (a) coacervates with CPK‐488, BSA‐TAMRA, HK‐647, and overlay. (b) coacervates with urease‐488, BSA‐TAMRA, GOX‐647, and overlay. Scalebars=20 μm. (c) Scheme of bioPIC process. (d) % Turbidity of the monomer, and polymer after reaction (along with unreacted monomer), filtered polymer to remove unreacted monomer on coacervation (*n*=3,±standard deviation). (e) The molecular weight distribution of PDMAEMA produced via bioPIC with a photograph of the turbid suspension after polymerization. (f) Scheme of the ERN‐PIC, (g) Time dependent changes in % Turbidity of in the presence, and absence of 0.4 U/mL ALP. (f) CLSM images of coacervates with segregated protein/enzymes, coacervates as black regions around urease‐488, BSA‐TAMRA, GOX‐647, and overlay. Insets are zoomed‐in images. [DMAEMA]=1.5 M, [ATP]=44 mM (bioPIC), 22 mM (ERN‐PIC). Scalebars=20 μm.

Next, to investigate if the enzyme catalysis happens within or out of coacervates, we observed the enzyme localization using CLSM. The catalytic activity of HRP present in the sample during the polymer synthesis by BioATRP is next tested. HRP catalyzes the production of fluorescent resorufin from its substrates Amplex Red in the presence of H_2_O_2_. We observed that the reaction happened preferentially within the droplets as observed by increased fluorescence within the droplets in comparison to solution. This confirms the compartmentalization of HRP and its maintained activity (Figure S11). By mixing bovine serum albumin (BSA) labelled with TAMRA with the polymer before coacervation, we observed a homogeneous, and consistent distribution in all coacervates (Figure S12). Similarly, Alexa Fluor 488‐labelled HRP, urease and CPK (HRP‐488, Urease‐488 and CPK‐488) and Alexa Fluor 647‐labelled HK and GOx (HK‐647 and GOx‐647) were uniformly entrapped by the coacervates (Figure 3a,b). Therefore, all tested proteins/enzymes were localized within the coacervates, and performed their reactions from within, ensuring fast and efficient action on the droplets.^[46]^

### ERN‐Polymerization‐Induced Coacervation (ERN‐PIC)

Our final aim was to achieve complete enzymatic control in a single pot integrating polymer synthesis, coacervation, and dissolution. First, we conducted a BioATRP reaction in the presence of 44 mM ATP, sufficient for coacervation (Figure 3c). The turbidity of the solution increased over time (Figure 3d). The small molecule DMAEMA, which cannot undergo coacervation with ATP, was gradually polymerized through BioATRP, resulting in biocatalytic polymerization induced coacervation (bioPIC).

The increasing multivalency, and charge density with polymer length promoted counterion ATP interaction, resulting in coacervation. Compared to monomer with same ATP concentration, the polymer exhibited ca. 5 times higher turbidity (Figure 3d). Monomer conversion was 72 %, and the polymers had size distribution of 20–40 kDa with low dispersity of 1.06 (Figure 3e, Table S1).Next, we performed ERN‐PIC, where HRP catalyzed monomer polymerization through BioATRP, which underwent coacervation in the presence of ATP, followed by dissolution through ALP‐mediated ATP hydrolysis to Pi (Figure 3f). As anticipated, an initial increase in turbidity was followed by a decrease, confirming the ERN‐PIC process (Figure 3g). In absence of ALP, only coacervation and no dissolution was observed.

Interestingly, coacervates formed during bioPIC were unable to take up labeled proteins added post‐coacervation (urease‐488, BSA‐TAMRA, or GOX‐647), suggesting a selective permeability barrier (Figure 3h). The partition coefficient was significantly lower with bioPIC coacervates (Figure S13). When coacervates were dissolved by removing ATP via spin‐filtering and reformed with fresh ATP, proteins localized within them (Figure S14). Further investigation with HRP‐488 revealed it localized outside the bioPIC coacervates, often adhering to their surfaces in semi‐circular patterns. This observation indicates exclusion from the dense internal network, consistent with the selective permeability of bioPIC coacervates. Small molecule dyes, such as resorufin, TAMRA, and Nile Red, localized within the coacervates, whereas labeled proteins such as GOX‐647, HK‐647, and BSA‐TAMRA were excluded, reinforcing the selective barrier. We hypothesize that in bioPIC, PDMAEMA polymerizes in the presence of ATP, with ATP acting as both a counterion and a crosslinker, creating a densely packed, interconnected network. This dense network restrict the diffusion of larger molecules like proteins while allowing smaller molecules, such as dyes, to pass through due to their size and the network's smaller voids. In contrast, when ATP is added after polymerization, the polymer chains are more flexible, creating a looser structure that permits both small and large molecules to diffuse. Thus, the order of ATP addition controls the coacervate‘s internal structure and the cargo it encapsulates. Similar findings with PDDA and ATP show ATP crosslinking controls RNA uptake.^[47]^ This ATP‐modulated molecular selectivity parallels our observations of enzyme exclusion or uptake depending on coacervate preparation methods (Figure S15). This selective permeability can enable controlled molecular exchange between macromolecules in solution and those within the coacervates, warranting further experimental and theoretical study. This study demonstrates the use of BioATRP to control coacervation in situ and provides a strategy for enzymatic control over coacervation in addition to environment and counterion‐based ERN. Additionally, it allows real‐time monitoring of the extent of coacervation with polymer lengths for new polymeric material, enabling the polymerization reaction to be stopped when the desired turbidity is observed. Thereafter, polymer lengths can be estimated.

## Conclusions

Our study introduces a novel approach to coacervate formation through in situ enzymatic polymer synthesis from small molecules in aqueous environments, closely mimicking natural polymerization processes. Unlike previous methods that rely on enzymes to alter external conditions such as pH, we employ an enzyme reaction network (ERN) to directly synthesize polymers while dynamically regulating their coacervation. This approach parallels the formation of intrinsically disordered proteins, where small amino acids polymerize to form protein and subsequently undergo coacervation, providing a simplified pathway to coacervate formation from small molecules without complex polymer synthesis in non‐aqueous solvents.

We developed bioPIC and ERN‐PIC systems to drive coacervation through polymer growth in the presence of ATP, regulated precisely by enzymatic activity. Moreover, we observed that the coacervates formed during the bioPIC were unable to take up any labeled proteins added from the outside, in contrast to post bioATRP coacervation. This suggests the formation of a highly selective permeability barrier within the coacervates based on the preparation method. To our knowledge, this level of dynamic control—where coacervation is intrinsically linked to polymerization—has not been previously achieved, marking a significant advance in integrating biological processes with synthetic materials under mild, aqueous conditions.

Our findings highlight the transformative potential of enzyme‐regulated coacervate systems to address complex challenges in biomimetic materials design, bridging biological pathways with synthetic polymer frameworks. Enzyme‐controlled coacervates have broad potential in various applications such as artificial cells and organelle models for studying cellular processes and producing biomolecules, targeted drug delivery and biosensing by releasing drugs or changing physical and optical properties in response to biomarkers. Additionally, coacervates can be used in smart materials for tissue engineering, soft robotics, and environmentally friendly material synthesis, offering bio‐inspired solutions and sustainable manufacturing alternatives.

## Supporting Information

The authors have cited additional references within the Supporting Information.[^48^, ^49^, ^50^]

## Author contributions

S.D. and A.B. designed the concept and idea. A.B. synthesized the polymer and performed antagonistic and orthogonal urease‐glucose oxidase, HK‐CPK enzyme kinetics, enzyme protein‐labeled dye imaging of coacervates, and bioPIC. S.S. and S.D. conducted the coacervation experiments with zero, one, and two enzymes. M.K., S.D. and S.S. carried out the FRAP experiment, and CLSM of coacervates. All authors analyzed the data and drafted the manuscript. All authors have given approval to the final version of the manuscript. There are no conflicts to declare.

## Conflict of Interests

The authors declare no conflict of interest.

1

## Supporting information

As a service to our authors and readers, this journal provides supporting information supplied by the authors. Such materials are peer reviewed and may be re‐organized for online delivery, but are not copy‐edited or typeset. Technical support issues arising from supporting information (other than missing files) should be addressed to the authors.

Supporting Information

## Data Availability

The data that support the findings of this study are available from the corresponding author upon reasonable request.
