# Supplementary material for: Enzymatic Reaction Network‐Driven Polymerization‐Induced Transient Coacervation
Source: Angew Chem Int Ed Engl. 2024 Dec 23;64(11):e202421620. doi: 10.1002/anie.202421620 (PMC11891636; doi:10.1002/anie.202421620)
Supplement: Supplementary file 1 — Supporting Information [file ANIE-64-e202421620-s001.pdf]

Angewandte Chemie
Eine Zeitschrift der Gesellschaft Deutscher Chemiker

Supporting Information

Enzymatic Reaction Network-Driven Polymerization-Induced Transient Coacervation

S. Sharma, A. Belluati, M. Kumar, S. Dhiman**

Enzymatic Reaction Network-driven Polymerization-induced Transient Coacervation

Contents

1. General Methods......S1
2. Experimental Protocols......S2
3. Synthesis......S3
4. Supporting Figures and Table......S4

1. General Methods

Chemicals and materials. All chemicals were procured from commercial sources and used without further purification. Spectroscopic-grade solvents were utilized for all spectroscopic measurements. AlexaFluor488-NHS-ester, AlexaFluor647-NHS-ester, and TAMRA-NHS-ester were purchased from Lumiprobe (Germany). HEPES, ATP, ADP, all enzymes, H_2O_2, Phosphocreatine disodium salt hydrate, glucose, urea, ethyl acetate, Nile red, Amplex red, and resorufin were purchased from Sigma Aldrich and used as received. The **enzymes used for the studies are:** HRP (horseradish): Type VI; Esterase from porcine liver; Alkaline phosphatase (*bovine intestinal mucosa*); Hexokinase (*from Saccharomyces cerevisiae*); Creatinephospho kinase (*from rabbit muscle*); and Urease (*from Canavalia ensiformis*).

Fluorescence Microscopy. Imaging of coacervates was performed on a Leica SP8 confocal laser scanning microscope, equipped with an HCX PL APO 63× NA 1.2 W CORR CS2 objective (Alexa Fluor 488: ex. 488 nm, em. 505–525 nm; Nile Red, resorufin, TAMRA: ex. 561 nm, em. 570–590 nm; Alexa Fluor 647: ex. 635 nm, em. 660–690 nm). Images captured with the software LAS X v5.0 and were optimized (brightness and contrast; applied evenly throughout the whole images) and analysed via FIJI v1.53.[48]

Fluorescence Recovery After Photobleaching (FRAP) study. FRAP was performed at room temperature employing confocal laser scanning microscope (Leica TCS SP8 / Leica Stellaris 5), and a laser excitation wavelength (λ_{exc}) of 514 nm. Leica oil-immersion objectives, specifically the HC PL APO CS2 63x with a numerical aperture (NA) 1.40 were used. The acquired confocal images were processed using LAS X (Leica) and FIJI software.

Microplate reader assays. OD_{600} kinetic assays were performed on a Clariostar Plus microplate reader (BMG Labtech) using Greiner transparent 96-well plates (24-well plates for yeast growth curves) with a flat bottom. Absorbance measurements were adjusted to correct for a 1 cm pathlength (ClarioStar software).

Decay constant determination. When necessary, the decay constant (k_{decay}) was determined according to Equation 1:

$$k = \frac{\ln(T_0 - C) - \ln(T(t) - C)}{t} \qquad \text{Equation 1}$$

Where: **k** is the decay constant, which quantifies the rate of decay, $\mathbf{T_0}$ is the initial turbidity value, **T(t)** is the turbidity at a given time t, **C** is the baseline turbidity. The averages of calculated k per concentration were compared with an unpaired t-test.

Determination of partition coefficient. Partition coefficient for various fluorescent molecules was determined using the formula: [49]

$$K_p = I_{\text{coacervates}} / I_{\text{outside}}$$

2. Experimental Protocols

- **Stock solutions** were prepared by adding appropriate amount of solid in MQ as follows: HK = 1000/mL; CPK = 3500 U/mL; Glucose = 2 M; PCr = 100 mM; ATP = 100 mM; ADP = 100 mM; GOx = 10 U/mL; Urease = 10 U/mL; Urea = 0.1 M.
- **HEPES-Br buffer preparations for the polymerization-** 50 mM HEPES buffer was supplemented with 100 mM NaBr (HEPES-Br).
- **HEPES buffer preparations for coacervation studies-** The appropriate amount of HEPES salt was added to MQ water to make the final 50 mM concentration and pH was adjusted to 7.3 by adding the required volume of 1M KOH.
- **Acetate buffer preparations for coacervation studies-** The appropriate amount of sodium acetate and acetic acid was added to MQ water to make the final 10 mM concentration and pH was adjusted to 4.5 by adding the required volume of 10N HCl.
- **Sample preparation for complex coacervates titrations-** To a polymer solution of 0.5 mM in 7.3 pH 50 mM HEPES solution, incrementally equivalents of ATP or ADP from their 100 mM stock solution in MQ water were added.
- **Sample preparation for kinetics with Alkaline Phosphatase –** To a polymer solution of 0.5 mM in 7.3 pH 50 mM HEPES solution, required units of ALP were added from its stock solution. To start the experiment, an appropriate volume of ATP was added.
- **Sample preparation for kinetics with Creatinine phosphokinase and Hexokinase -** To a polymer solution of 0.5 mM in7.3 pH 50 mM HEPES solution, required units of CPK, HK, glucose was added from their stock solutions. To start the experiment, appropriate volume of ADP and PCr, was added.
- **Sample preparation for kinetics with esterase and urease -** To a polymer solution of 1.25 mM (Batch 3) in 4.5 pH 10 mM acetate buffer, the appropriate volume of ATP was added followed by the addition of required units of esterase and urease. To start the experiment, respective substrates ethyl acetate and urea was added.
- **Sample preparation for pKa value:** A 0.5 mM polymer solution in an appropriate volume of 0.5 M HCl was titrated with continuous 0.5 µL increments of 0.5 M NaOH solution."
- **Solution Preparations for Microplate reader assays-** The final volume in each well was 200 µL (with HEPES). For ALP assays, **P1** was used, otherwise **P2**. Final concentrations were: [PDMAEMA] = 0.5 mM (1.25 mM for esterase-urease assay), [ATP] = 5 mM, ALP = 2, 5 or 10 U mL^{-1}, [ATP] =[ADP] = 5 mM (8 mM for esterase-urease assay,) CPK = 20U, HK = 10

U, [Pcr] = 7.5 x 10^{-3} M, [Ethyl acetate] = 240 mM, Esterase = 200 U, Urease = 285 U, [urea] = 24 mM. For urease esterase kinetics **P3** was used.

- **Processing of UV/vis absorbance data into turbidity formula -** The absorbance at 600 nm is converted to % Turbidity by using the following equation: % Turbidity = $100 - 100 \times 10^{-A}$
- **Protein labelling preparations-** 1 mM stock solutions of TAMRA-NHS ester, AlexaFluor488-NHS ester and AlexaFluor647-NHS ester were prepared in anhydrous DMSO. Stock solution of 5 mg mL^{-1} of urease, GOX, HRP and BSA were prepared in HEPES pH 8.3, CPK and HK were instead kept at pH 7.0 in 3500 U/mL and 1000/mL, respectively. To 455 µL of protein solution, 5 µL of dye were added and let react overnight at 4°C. Then, they were purified via a 10 kDa MWCO spin filter tube (Millipore), centrifugating at 12000 x g, 10 min, and resuspended in HEPES pH 7.4.
- **Polymer characterization -** ^{1}H NMR spectroscopy was conducted on a Varian Unity 300 MHz spectrometer operating at 300 MHz. 200 µL of a sample were diluted in 600 µL D_2O. The ratio between the integral of the vinyl protons of the monomer with the methylene protons of the backbone was used to calculate the monomer conversion according to a published protocol.[50] The GPC analysis was conducted with water as an eluent on a PSS liquid chromatography system equipped with a PSS G1362A refractive index detector (λ = 633 nm) and using 1 guard column and 2 identical PSS Suprema Linear M columns (5 µm bead size, hydroxylated methacrylate-based bed) in series operating at 25 °C. Water with 0.1 M $NaNO_3$ and 0.05% w/v NaN_3 was employed as the mobile phase at a flow rate of 1 $mL \cdot min{-1}$. The system was calibrated using pullulan standards (180 to 708000 Da). All samples were filtered through 0.45 µm Teflon filters prior to injection.
- **CLSM imaging for dyes encapsulation and resorufin production**
  - Samples were placed in Nunc® Lab-Tek® 8-well chamber slides (Thermo Fisher), 12.5 µl of sample in 200 µl HEPES, and 10 equivalents ATP.
  - *Resorufin production*-Coacervates were formed with ATP, and then 10 µl of a 1 mm stock solution of Amplex Red and 10 µl of H_2O_2 0.03 vol% were added right before imaging.
  - *Protein entrapment*-Depending on the combination, 5 µl of BSA-TAMRA, HRP-488, CK-488, urease-488, HK-647 and GOX-647 were added to the PDMAEMA solution before ATP and coacervation or, in one control experiment, first coacervates were formed and then the proteins added to the chamber wells.
  - Imaging of bioPIC coacervates **-** In order not to dilute ATP in fresh HEPES, 200 µL of the suspension were instead pre-mixed with the labelled proteins in an Eppendorf tube, and 20 µL of the mixture were placed onto a round glass microscope slide and covered with a round coverslip.
- **Spin Filtering** – In order to remove either unreacted DMAEMA or ATP from BioPIC coacervates, 250 µL x 2 of the coacervate suspension was diluted in 250 µL HEPES, centrifuged for 10 minutes at 10000 RCF in Vivaspin centrifugal concentrators (Cytiva, 10000 MWCO), resuspended in additional 500 µl HEPES, centrifuged and then resuspended in the original 250 µL volume (HEPES). This allowed to remove small molecules. The coacervates where re-formed by adding fresh ATP.

3. Synthesis of PDMAEMA by BioATRP

The procedure was adapted from previous work.[35] In batch one, at least 1000 µL of DMAEMA was deprotected by passing it through a short basic alumina plug (vial A). An excess of sodium ascorbate (12 mg), ethyl-α-bromophenylacetate (EBPA, 1.3 µL), and DMSO (200 µL) were dissolved in 0.8 mL of HEPES-Br (vial B) for a target degree of polymerization (DP) of 400. HRP (3 mg) was dissolved in 1 mL of HEPES-Br in a round-bottom flask and all solutions were sparged with Argon gas for 30 minutes. The contents of vial B were added to the flask at 37°C and allowed to equilibrate for 5 minutes. Then, 500 µL of vial A was added. In batch 2, DP and volume were kept constant, but concentrations of DMSO, monomer and initiator were halved.

The reaction proceeded (under Argon) for 3 hours at 37 °C with stirring at 400 rpm. The two batches produced polymers **P1** and **P2**, with remarkable close in polymer weight and dispersity, hence they were used interchangeably in various experiments.

BioPIC - For BioPIC, the conditions of batch 2 were used, with the addition of 44 mM (12 eq. ATP assuming 100% conversion, [DMAEMA]:[ATP] 1:0.015) promoting coacervation as the polymer chains grew. The monomer turbidity was also measured with the same ATP concentration.

• ***ERN-PIC:*** the same conditions were applied for ERN-PIC, with the only differences being a lower ATP concentration (22 mM, 6 eq.), sufficient to promote coacervation, and 0.4 U/mL ALP (none in the control), promoting coacervation and dissolution as the polymer chains grew. To measure the evolution of turbidity, 100 µL of sample were taken at t=0, 30, 60 minutes and then every hour for 5 hours and immediately measured at the microplate reader; At t = 180 minutes, the vessels were opened and air allowed into the solution, quenching the polymerization, but not ALP.

5. Supplementary Tables and figures

	Monomer conversion (%)	M_n	Đ	DP
**P1**	61%	35690	1.1	~227
**P2**	57%	31140	1.1	~198
**BioPIC**	72%	45430	1.06	~289

Table S1: monomer conversion, molecular weight, dispersity index (Đ) and degree of polymerization (DP) of the two polymer batches used in this study.

S.No.	ADP concentration (mM)	k_{decay} (min $^{-1}$)
1.	4	0.21±0.1
2.	5	0.244 ±0.8

Table S2: Average decay constant at different concentrations of ADP.

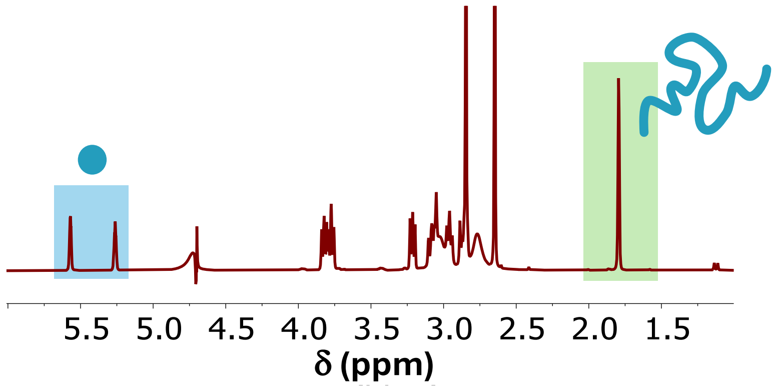

Figure S1:^{1}H-NMR spectra of PDMAEMA recorded in D_2O. Blue region corresponds to monomers, green region corresponding to polymer.

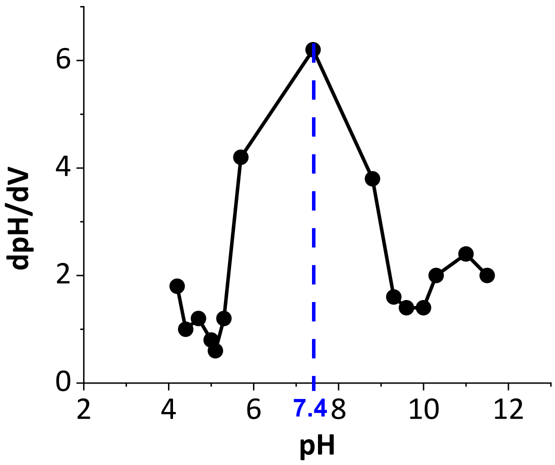

Figure S2: Plot of first derivative of pH with respect to volume of base added to calculate pKa.

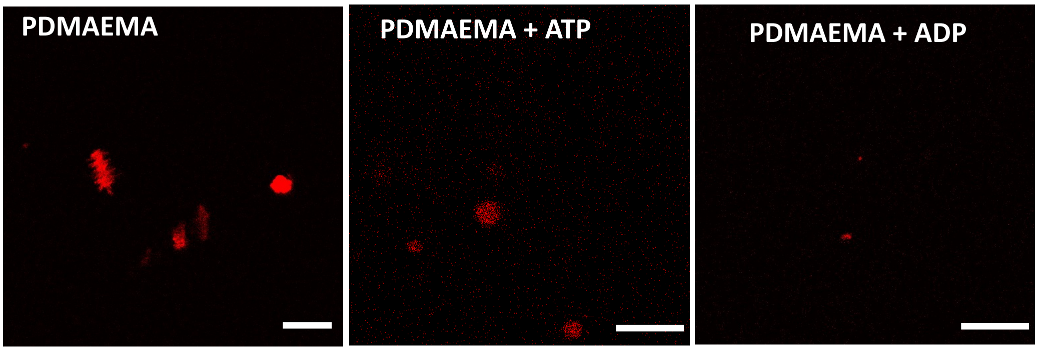

Figure S3: CLSM images of PDMAEMA alone, PDMAEMA with ATP and PDMAEMA with ADP. PDMAEMA] = 0.5 mM, [ATP] = [ADP] 5 mM, 50 mM HEPES, pH 7.3, and [Nile red] = 0.5 mol %. Scale bar = 10 µm

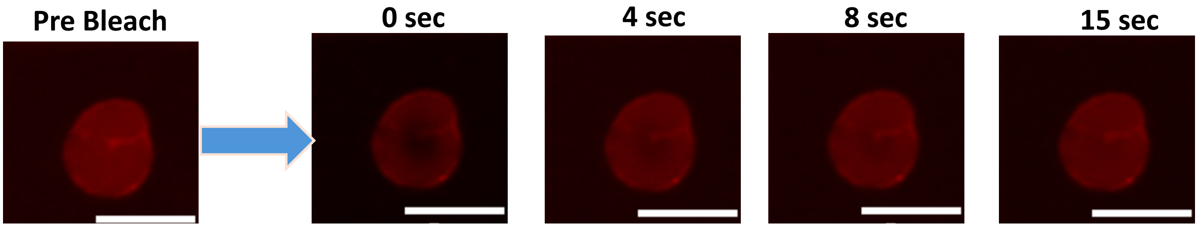

Figure S4: Expanded CLSM images obtained during FRAP of complex coacervates of PDMAEMA with ATP. [PDMAEMA] = 0.5 mM, ATP = 5mM, [Nile red] = 0.5 mol %. HEPES = 50 mM, pH 7.3. Scale bar = 5μm.

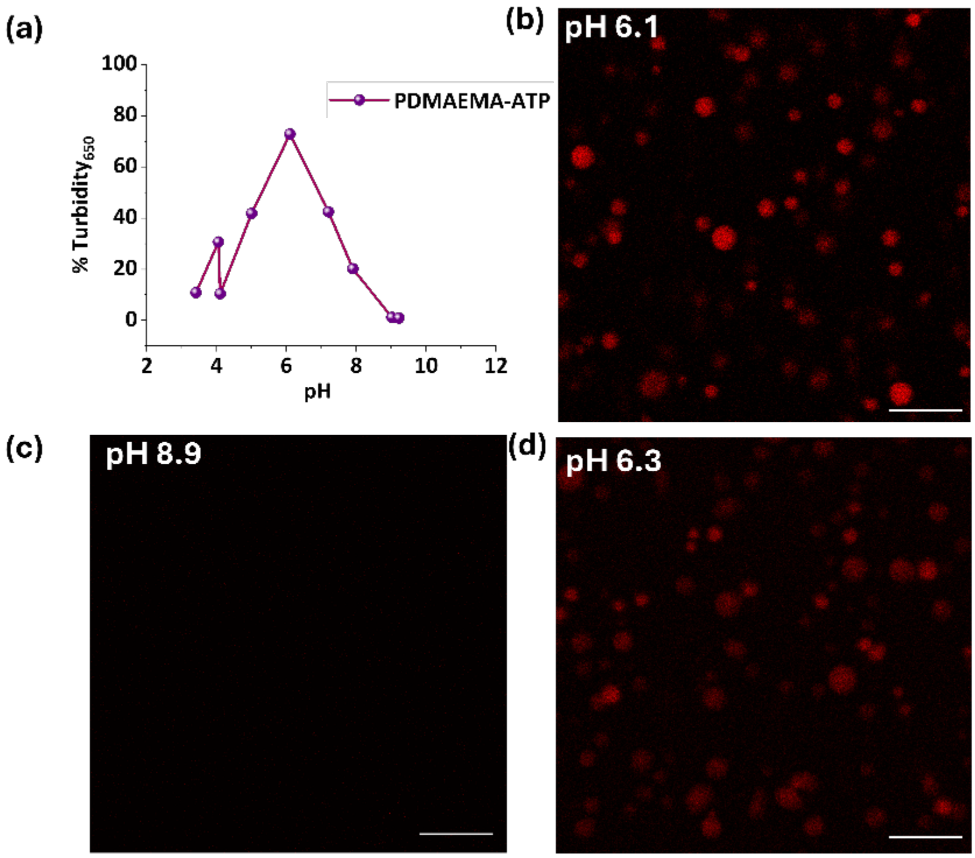

Figure S5: pH-dependent (a) % Turbidity (b-d) CLSM images of complex coacervates of PDMAEMA with ATP. [PDMAEMA] = 0.5 mM, ATP = 5mM, Scale bar is 10 μm.

Note: The reversibility, and responsiveness of this coacervation were confirmed by changing the pH of solution increasing the pH of the solution to 9 by using 0.5 M NaOH, which resulted in a decrease in turbidity, and the disappearance of coacervates. This effect is attributed to the deprotonation of PDMAEMA, leading to a loss of electrostatic interactions between PDMAEMA, and ATP. Subsequently, when the pH was lowered back to 6 by using 0.1 M HCl, an increase in turbidity was observed, and coacervates reappeared.

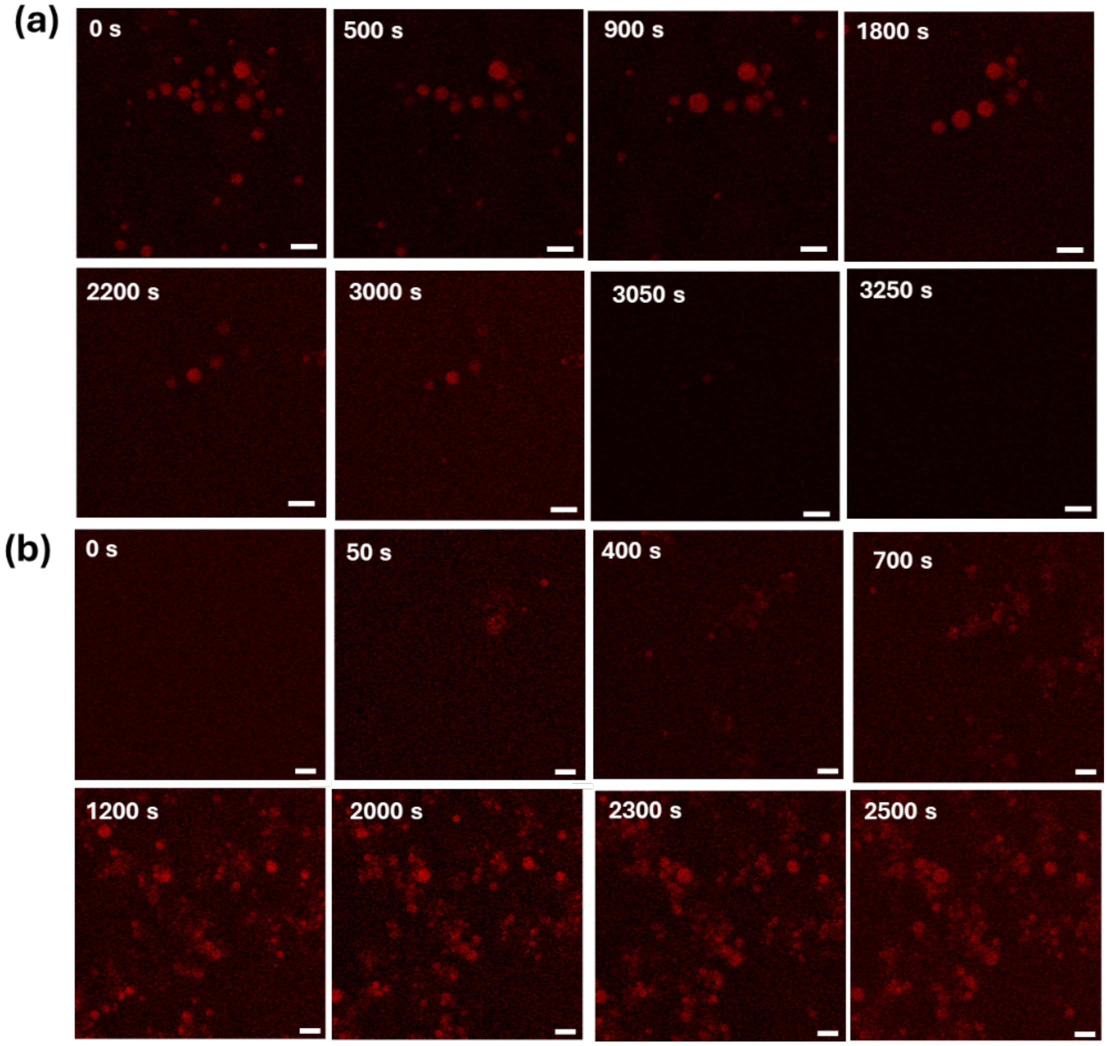

Figure S6: Time-dependent CLSM images with pH-modifying enzymes (a) Urea-Urease (b) ethyl acetate-Esterase (within the same sample). [PDMAEMA] = 1.25 mM, [ATP] = 8 mM, Esterase = 200 U/mL, Urease = 285 U/mL, [ethyl acetate] = 240 mM, [urea] = 24 mM, and 4.5 pH, 10 mM acetate buffer. Scale bar is 5 μm. [Resorufin] = 0.2 mol %.

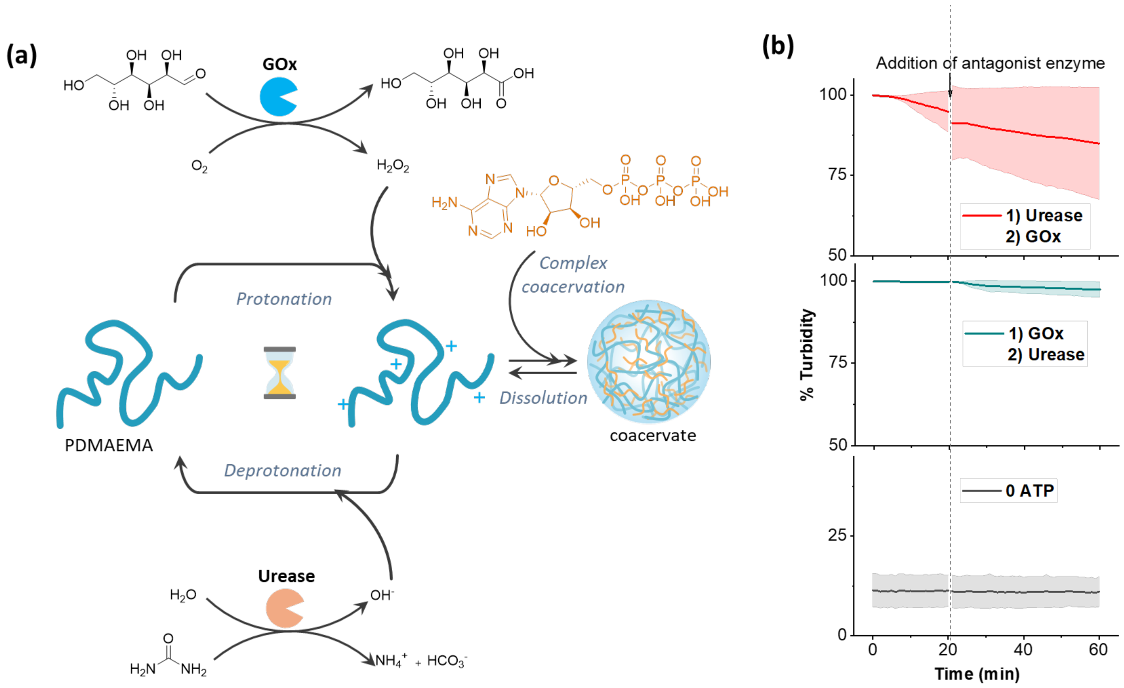

Figure S7: Enzymatically mediated stimuli responsiveness of the coacervates. (a) schematic, (b) Kinetics of the pH-modifying enzymes (urease and GOx). The antagonist enzymes (GOx to urease, urease to GOx) were added 21 minutes after the start of the reaction. [PDMAEMA] = 0.5 mM, [ATP] = 6 mM (12 Eqv.) GOx = 10 U/mL, Urease = 10 U/mL, [Glucose] = 0.1 M, [urea] = 0.1 M, and 7.3 pH, 50 mM HEPES. N = 3, ± standard deviation.

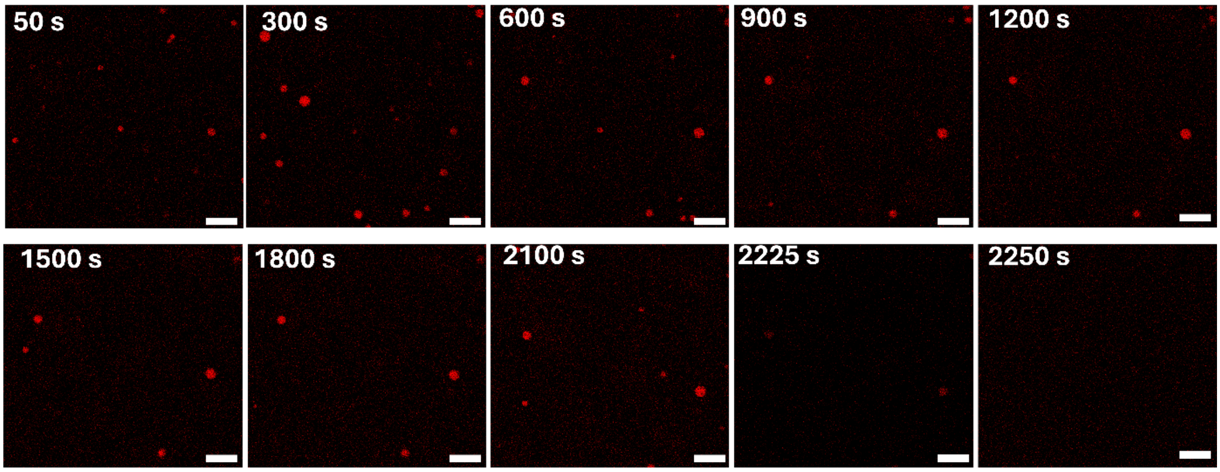

Figure S8. Time-dependent CLSM images with 10 U/mL ALP concentration; [PDMAEMA] = 0.5 mM, [ATP] = 5 mM, 50 mM HEPES, pH 7.3, and [Nile red] = 0.5 mol %. Scale bar = 10 µm.

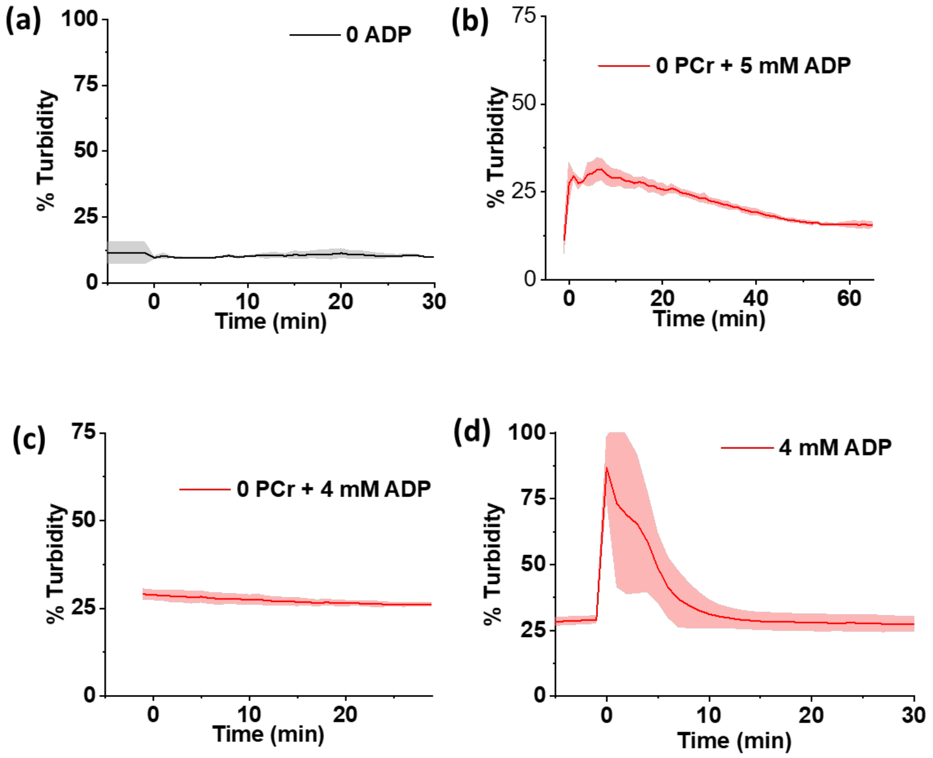

Figure S9: Kinetics of turbidity measurements of blank for CPK and HK kinetics, (a) without ADP, (b) without PCr and 4 mM ADP, (c) without PCr and 5 mM ADP, and (d) 4 mM ADP. [PDMAEMA] = 0.5 mM, CPK = 20U/mL, HK = 10 U/mL, 50 mM HEPES and pH 7.3. N=3, ± standard deviation.

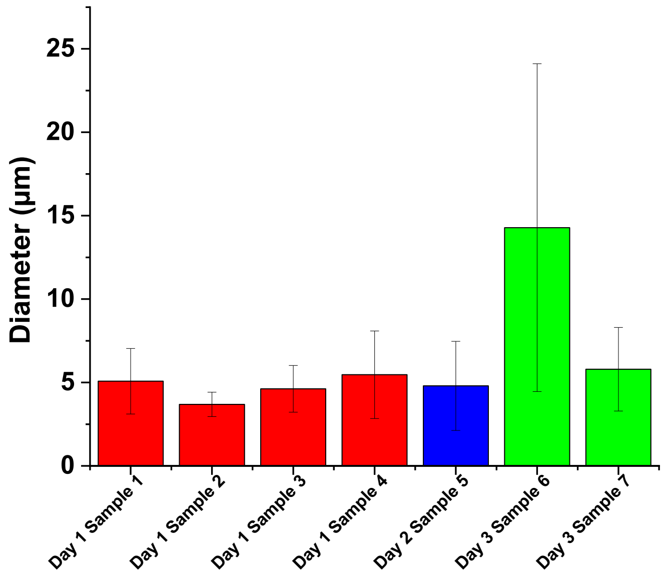

Figure S10: Size distribution of coacervate samples prepared at different days and at different times.

Note : When we increased the concentration of ADP to 5mM, we observed an increased lifetime for the existence of coacervate from 7.8±2.4 to 11.7±2.9 minutes, however, the

average decay constant k_{decay} for 4 and 5 mM ADP show statistically non-significant differences (0.21±0.1 min-1 and 0.244 ±0.8 min-1, respectively, p = 0.7), due to the high coacervate population variability in size and number. This variability indicates that while increased ADP enhances transient coacervate stability, it fundamentally does not impact the decay rate (Table S2).

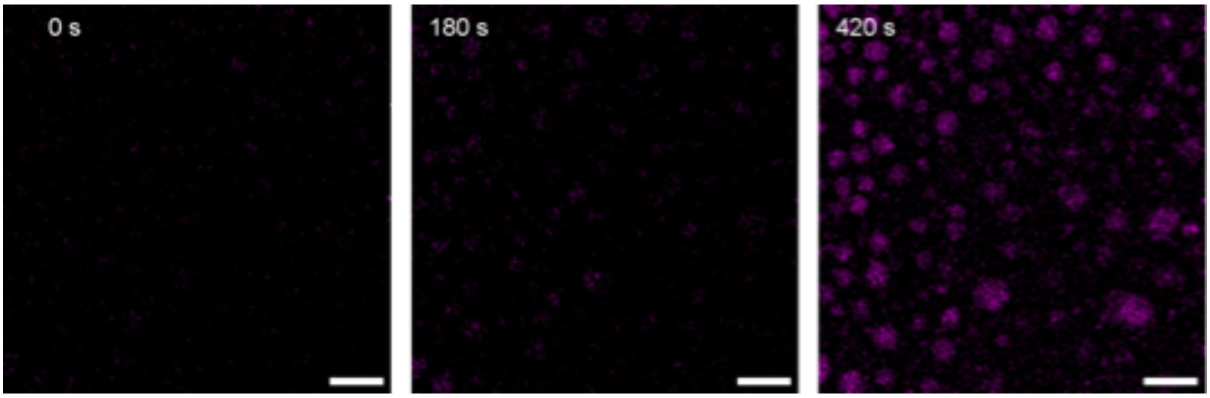

Figure S11: Timepoints of CLSM micrographs of resorufin synthesis from Amplex Red and H_2O_2 by HRP encapsulated within the coacervates. Scalebars: 20 µm.

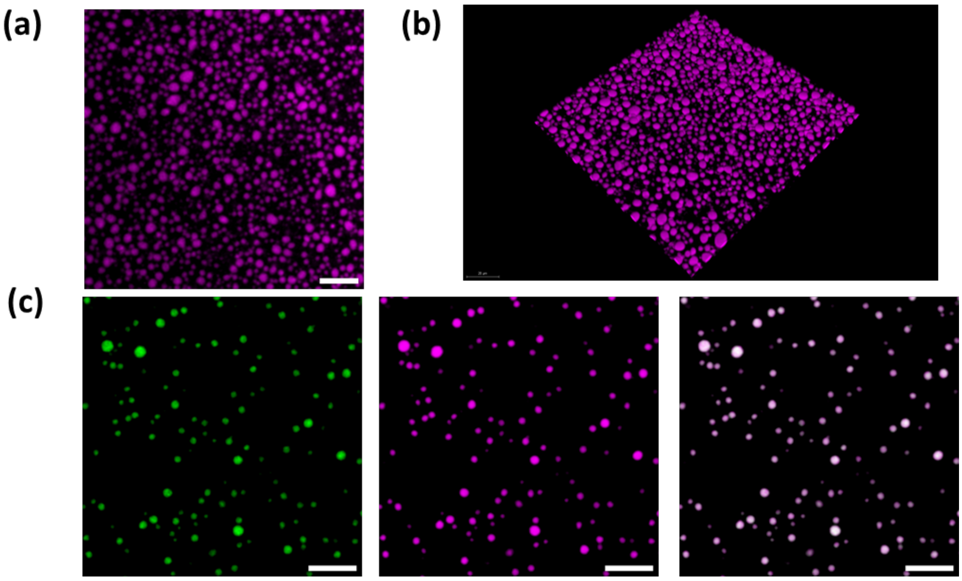

Figure S12: (a) CLSM imaging of PDMAEMA-ATP coacervates with BSA-TAMRA, (b) 3D Z-stack fluorescence CLSM images of PDMAEMA-ATP coacervates with BSA-TAMRA, (c) Complex Coacervates of PDMAEMA-ATP with HRP-488 (green), BSA-TAMRA (magenta), and overlay. Scalebars: 20 µm.

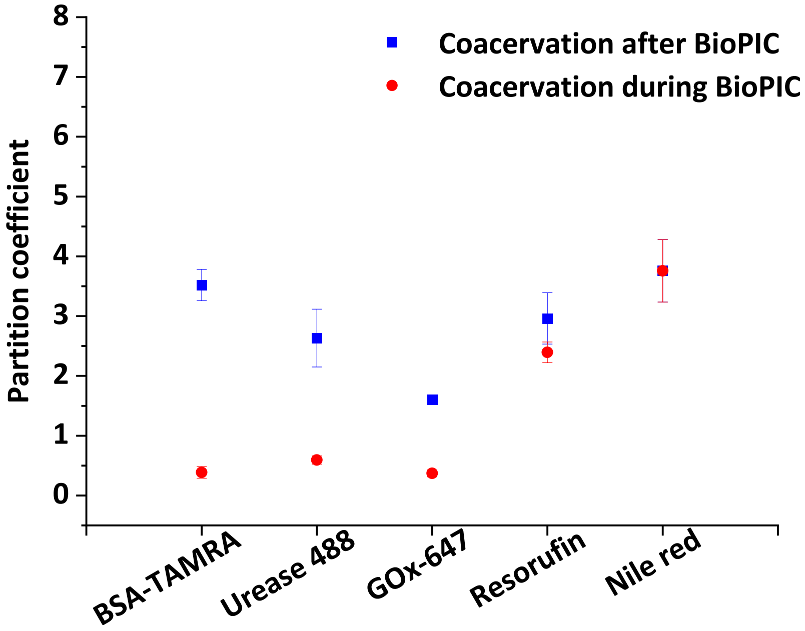

Figure S13. Partition Coefficients of different enzymes during and after BioPIC coacervation.

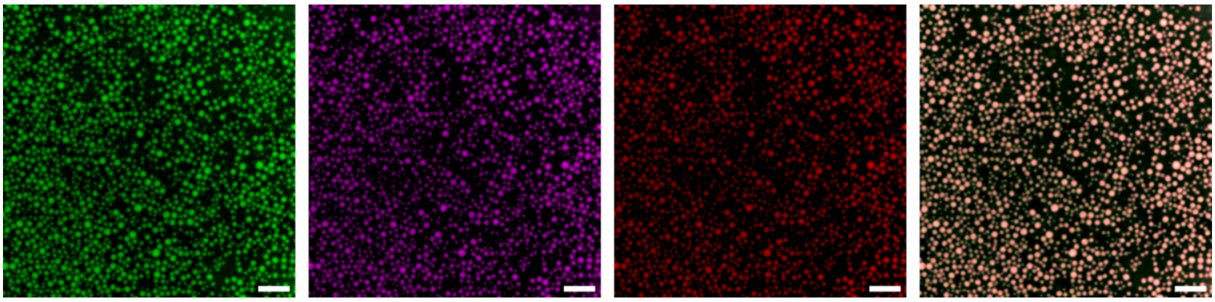

Figure S14. CLSM micrographs of coacervates with labelled proteins added to coacervates produced via BioPIC, redissolved (via spin filtering) and then reformed. From left to right: CK-488, BSA-TAMRA, HK-647. Scalebars: 20 µm.

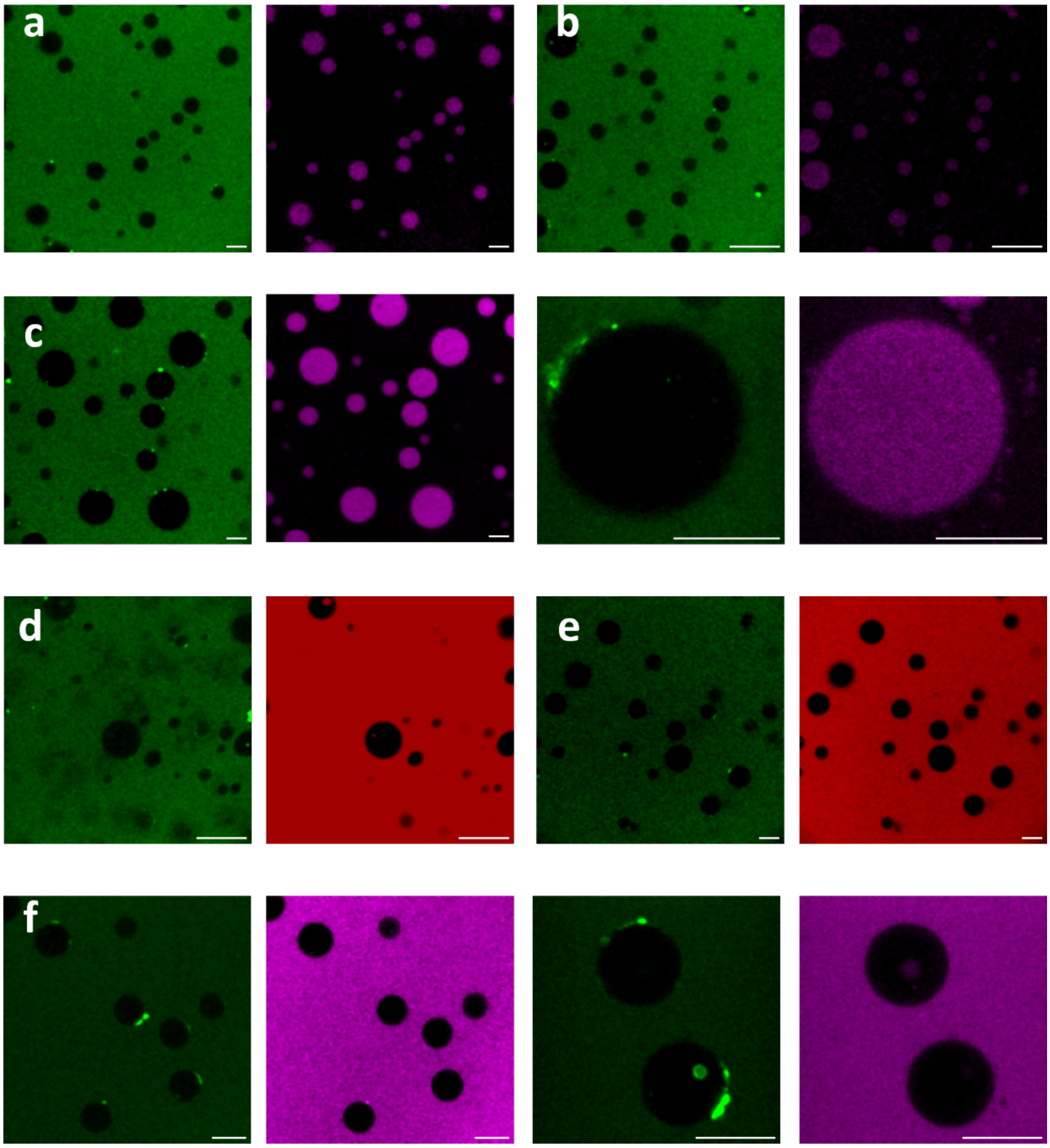

Figure S15. CLSM imaging of BioPIC coacervates produced HRP-Alexa Fluor 488 (green) and other dye molecules or labelled proteins. (a) Resorufin, (b) TAMRA (c) Nile Red, (d) HK-Alexa Fluor 647, (e) GOX-Alexa Fluor 647, (f) BSA-TAMRA. Scalebars: 20 μm.
